# Emerging role of the interleukin (IL)-33/ST2 axis in gut mucosal wound healing and fibrosis

**DOI:** 10.1186/1755-1536-5-18

**Published:** 2012-10-14

**Authors:** Loris R Lopetuso, Franco Scaldaferri, Theresa T Pizarro

**Affiliations:** 1 Department of Pathology, Case Western Reserve University School of Medicine, 2103 Cornell Road, WRB 5534, Cleveland, OH, 44106, USA; 2 Department of Internal Medicine, Gastroenterology Division, Catholic University of Rome, Policlinico Universitario Agostino Gemelli, Largo Agostino Gemelli 8, Rome, 00168, Italy

**Keywords:** IL-33/ST2 axis, IL-1Family, Alarmin, Inflammatory bowel disease, Mucosal healing, Epithelial restoration/repair, Intestinal fibrosis, Tumorigenesis

## Abstract

Interleukin (IL)-33 (IL-1F11) is the newest member of the IL-1Family of cytokines and has been best characterized as a potent inducer of T helper (Th)2 immune responses. Increasing evidence, however, indicates that IL-33 also represents an important mediator of mucosal healing and epithelial restoration and repair. As such, IL-33 follows the trend of several innate-type cytokines, including members of the IL-1Family (for example, IL-1α, IL-1β, and IL-18), that possess dichotomous roles of inducing a potent proinflammatory response, while also promoting protection and the return to immune homeostasis. This dual function is best depicted in the gut mucosa and is dependent upon the immunological/genetic status of the host and/or the type and phase of the ongoing inflammatory process. IL-33 has also been described as a prototypic ‘alarmin’ that has the ability to signal local, innate immune responses of trauma or infection in an effort to mount an effective, physiologic inflammatory reaction to induce mucosal healing and restore normal gut equilibrium. Finally, several recent studies have reported the role of IL-33 during fibrogenesis as fibrosis is commonly thought to occur as the end stage of dysregulated wound healing wherein chronic tissue damage is paired with uncontrolled activation of mesenchymal cells. Taken together, aside from its established function of promoting potent Th2 immune responses, IL-33 is emerging as an important cytokine for the induction of mucosal healing and restoration of intestinal homeostasis, as well as playing a central role in fibrosis and wound repair. The present review will focus on what is currently known regarding IL-33’s role in gut mucosal wound healing and fibrosis, as well as touch on its potential contribution to tumorigenesis and GI-related cancer, an alternate outcome of dysregulated epithelial proliferation.

## Review

### Introduction

The role of interleukin (IL)-1 and its related cytokine family members is well established in the pathogenesis of several autoinflammatory and chronic immune disorders, including inflammatory bowel disease (IBD) [1]. IL-33, also known as IL-1F11, represents the most recently identified member of the IL-1Family, which also includes the classic cytokines IL-1α, IL-1β and IL-18. IL-33 is a protein with a dual function that can act both as a signaling cytokine as well as an intracellular nuclear factor, originally reported to be preferentially localized to the high endothelial venules of human tonsils, Peyer’s patches and lymph nodes [2]. More recent evidence suggests that IL-33 is widely distributed throughout various organ systems in the body, primarily expressed in nonhematopoietic cells, including fibroblasts, adipocytes, smooth muscle cells, endothelial cells, bronchial and intestinal epithelial cells (IEC) [3-5], but is also present in cells of hematopoietic origin, particularly in restricted populations of professional antigen-presenting cells, such as macrophages and dendritic cells [5].

IL-33 exerts its biological effects through binding to its receptor, IL-1 receptor-like 1 (IL1RL1), also known as ST2 [5,6]. In the presence of IL-33, ST2 pairs with its coreceptor, the IL-1 receptor accessory protein (IL-1RAcP), and allows signaling through mitogen-activated protein kinase and NF-κB [5,7]. ST2 has been reported to be constitutively expressed in mast cells, as well as T helper (Th)2 lymphocytes in both mice and humans [5,8,9].

Initially, IL-33 was associated with the development of Th2 immunity, based on the expression of its cell-bound receptor, ST2L (IL-1R4), in polarized Th2 lymphocytes and its ability to induce the production of Th2 cytokines (for example, IL-4, IL-5 and IL-13), in both *in vitro* and *in vivo* systems [5]. Moreover, exogenous administration of recombinant IL-33 in mice led to Th2-mediated immune responses, inducing eosinophilia, splenomegaly, goblet cell hyperplasia and mucus production at mucosal surfaces, and increased serum levels of IL-5 and IgE [10]. IL-33 has been shown to be involved in airway inflammation [11-13], allergic reactions [14-16], and rheumatic diseases [17,18], and also to reduce the development of atherosclerosis through the induction of IL-5 [19]. IL-33, however, has also been described to exacerbate arthritis, widely considered a Th1/Th17-mediated pathology [17,18].

In regard to its role in the gastrointestinal (GI) tract, IL-33 appears to play a critical role in maintaining normal gut homeostasis. IL-33 has been shown to enhance mucosal defenses against intestinal parasites and bacteria, as described for *Toxoplasma gondii*[20], *Pseudomonas aeruginosa*[21] and *Leptospira*[22] infection, indicating a primary role of protection. Elevated expression of IL-33 has also been reported in the inflamed mucosa of IBD patients, mainly in ulcerative colitis (UC), and to a lesser extent, in Crohn’s disease (CD) patients [23-26]. Although these initial studies suggest a pathogenic role for IL-33 in IBD, the specific localization of subepithelial myofibroblasts (SEMFs) below ulcerative lesions in UC, but not in CD, patients, indicate the potential role for IL-33 in wound/ulcer healing [24].

In fact, aside from its elicitation of mounting a potent Th2 immune response, emerging evidence supports the possible role of the IL-33/ST2 axis in modulating wound healing and fibrosis. Fibrosis is commonly believed to represent the end stage of the repair process during chronic and/or relapsing tissue damage, wherein inflammatory cell infiltration and resident fibroblast activation persists, while the reparative ability of mesenchymal stem cells is diminished. Heart failure and cardiac fibrosis have been reported to be associated with the production of IL-33 from cardiac fibroblasts [27], while ST2 and transforming growth factor beta (TGFβ) have been shown to be increased in a model of bleomycin-induced lung fibrosis [28]. Additionally, in a mouse model of liver fibrosis induced by carbon tetrachloride, administration of an ST2-Fc fusion protein was found to increase Th2 cytokine production and enhance hepatic fibrosis [29].

Taken together, there is clear evidence of the IL-33/ST2 axis in maintaining normal gut homeostasis, particularly in promoting mucosal wound healing and repair. When dysregulated, this important ligand-binding pair can also play a critical role in the progression of chronic inflammation and fibrosis, leading to such GI-related disorders as IBD. The purpose of this review is to explore the role of IL-33 in modulating epithelial repair, mucosal healing, and fibrosis in the GI tract during normal gut homeostasis and in the setting of chronic intestinal inflammation. We will also touch upon the potential link between IL-33/ST2 in the process of tumorigenesis and GI-related cancer, an alternate outcome when uncontrolled and/or dysregulated epithelial proliferation occurs.

### IL-33 in epithelial restoration/repair, mucosal healing, and maintenance of normal gut homeostasis

The GI tract, with its epithelial barrier consisting of a total surface area of approximately 200 m^2^, is man’s most widely exposed organ system to the external environment. The intestinal barrier represents a functional unit responsible for two main tasks crucial for survival of the individual: allowing nutrient absorption, and defending the body from penetration of unwanted, often dangerous, macromolecules. The gut mucosa is a multilayer system consisting of an external ‘anatomical’ barrier and an inner ‘functional’ immunological barrier. Commensal gut microbiota, a mucous layer, and the intestinal epithelial monolayer constitute the anatomical barrier. The deeper, inner layer consists of a complex network of immune cells organized in a specialized and compartmentalized system known as ‘gut-associated lymphoid tissue’ or GALT. GALT represents both isolated and aggregated lymphoid follicles and is one of the largest lymphoid organs, containing up to 70 % of the body’s total number of immunocytes, and is involved in responding to pathogenic microorganisms and in providing immune tolerance to commensal bacteria. The ability of GALT to interact with the luminal antigens rests on specific mucosal immune cells (that is, dendritic cells and M cells), primarily localized to Peyer’s patches within the ileum, that are intimately positioned at the mucosal-environmental interface and internalize microorganisms and macromolecules. These specialized immune cells have the ability to present antigen to naïve T lymphocytes, which subsequently produce cytokines and activate mucosal immune responses, when needed. The interaction of these components sustains the maintenance of the delicate equilibrium of intestinal homeostasis. Many factors can alter this balance, including alterations in the gut microflora, modifications of the mucus layer, and epithelial damage, leading to increased intestinal permeability and translocation of luminal contents to the underlying mucosa. The integrity of these structures is necessary for the maintenance of normal intestinal barrier function and dysregulation of any of the aforementioned components have been implicated not only in the pathogenesis of IBD, but many other GI disorders, including infectious enterocolitis, irritable bowel syndrome, small intestinal bowel overgrowth, and allergic food intolerance [30-32].

Emerging evidence indicates a paramount role for IL-33 in the maintenance of gut mucosal homeostasis. Like IL-1α, IL-33 appears to serve as a dual function protein. Full-length, unprocessed IL-33 contains a nuclear localization sequence and a DNA-binding domain [33] that has been shown to be constitutively localized to the nucleus of epithelial, as well as endothelial, cells [4]. Under normal conditions, IL-33 can act as an intracellular nuclear factor [2] that, upon inflammatory stimuli, is released from the cell through a currently unknown mechanism and behaves as a functional, secreted cytokine [33]. One of the earliest observations regarding the biological activity of IL-33 is its ability to promote epithelial proliferation and mucus production [5], which are obvious functions involved in epithelial restitution and repair, as well as overall mucosal wound healing and protection. In addition, and similar to IL-1α, increasing evidence indicates that IL-33 can function as a prototypic ‘alarmin’, passively released upon cellular damage, stress, or necrosis, and able to serve as a danger signal/alarmin to alert the immune system of a local threat, such as trauma or infection [4,34-36]. In this setting, IL-33 has the ability to signal local, innate immune responses in an effort to mount an effective, physiological inflammatory reaction in order to restore normal gut homeostasis.

### IBD pathogenesis and the IL-33/ST2 axis

In IBD, characterized by chronic, relapsing inflammation with global epithelial barrier dysfunction and persistent leakiness [37,38], efficient mucosal healing is one of the most prominent clinical needs to be addressed and goals to be achieved in order for patients to maintain long-term remission [39]. On the other hand, the repair process involves the orchestration of several different intestinal mucosal cell populations that, during chronic and persistent tissue damage, may result in the generation of excessive connective tissue deposition and the development of fibrosis.

It is now well established and confirmed by several groups that increased IL-33 expression is associated with IBD when compared to healthy controls, particularly in UC patients [23-26]. Gut mucosal expression of IL-33 is primarily localized to nonhematopoietic cells, particularly IEC [23,25,26] and myofibroblasts [24]. In addition, *ex vivo* studies on isolated intestinal mucosal cell populations and immunolocalization on full-thickness intestinal tissues show that IL-33 is also expressed by a wide variety of cell types [24,25,40], such as fibroblasts, smooth muscle cells, endothelial cells [5,19], and adipocytes [3,25]. In active UC, IL-33 is localized to, and potently expressed by, IEC, as well as infiltrating lamina propria mononuclear cells, belonging to the monocyte/macrophage and B cell lineages [23-25]. It has also been originally reported by Kobori *et al*. [24], and later confirmed [40], that IL-33 is expressed in activated SEMFs situated below ulcerative lesions in UC, but not in CD, patients supporting a potential functional role for IL-33 in ulcer/wound healing, which may be different in UC compared to CD.

Similar to IL-33, its receptor, ST2, is also increased in the intestinal mucosa of IBD patients [23,25]. Importantly, the intestinal tissue expression pattern of ST2 is different in healthy mucosa compared to that found in chronically inflamed IBD patients, wherein ST2 is abundantly expressed in macroscopically noninflamed colon epithelium, while during chronic inflammatory processes characterizing either UC or CD, its expression is lost/decreased and redistributed [6]. This epithelial-derived tissue expression for ST2 appears to be IBD-specific since non-specific colitides (for example, diverticulitis and infectious colitis) do not present with this same expression pattern [25].

Taken together, considering the potential role of IL-33 in promoting mucosal protection, as well as its tissue distribution in IBD, it is tempting to speculate that the primary role for IL-33 is, in fact, to induce epithelial restitution and repair and mucosal healing. In addition, further analysis has shown that the ST2 variant for which expression is altered in the epithelium of IBD patients is ST2L, IL-33's signaling transmembrane receptor [6,23]. As such, it is possible that impaired epithelial ST2L expression, specifically in IBD patients, may represent an inherent epithelial defect or a negative feedback response to chronic exposure of elevated IL-33 concentrations. One cannot rule out, however, that IL-33 may have pathogenic, as opposed to protective, effects on the epithelium, as well as consider its effects on mucosal immune cell populations to induce the infiltration of innate cells, specifically neutrophils and eosinophils, and mount potent Th2, Th17, and potentiate Th1 inflammatory responses. In fact, the dichotomous role of IL-33 as well as other innate-type cytokines, including several members of the IL-1Family, have been best characterized in the intestine, where they can possess both protective and proinflammatory functions, depending upon the immunological status of the host and/or the type and phase of the ongoing inflammatory process [6,41]. Investigation has turned to the use of animal models of IBD to mechanistically address the specific role of the IL-33/ST2 axis in IBD (summarized in Table 1) [17,18,27,28,42-47].

**Table 1:** Role of IL-33/ST2 axis in animal models of tissue repair and fibrosis

**Target Organ/ System**	**Animal Model**	**Model Manipulation**	**Proposed Role of IL- 33 / ST2**	**Postulated Mechanism**
**Intestine**				
**Chemically-induced barrier disruption, follwed by intestinal inflammation**	**Acute DSS colitis**	**Genetic deletion of IL-33**	**Pathogenic**	**Neutrophil recruitment**[42]
		**Exogenous IL-33 administration**	**Pathogenic**	**Neutrophil recruitment**[43,44]
	**Chronic DSS colitis**	**Exogenous IL-33 administration during recovery phase**	**Protective**	**Th1→Th2 switch: improvement of bacterial clearance**[44]
**Chemically-induced ThI- mediated intestinal inflammation**	**TNBS colitis**	**Exogenous IL-33 administration**	**Protective**	**Th1→Th2 switch: expansion of Treg population**[45]
**Spontaneous, chronic intestinal inflammation**	**SAMP1/YitFc mice**	**Blockade of IL-33 by anti-ST2 antibody administration**	**Pathogenic**	**Activation of pathogenic IL-17-producing macrophages**
		**Blockade of IL-33 by anti ST2 antibody administration**	**Pathogenic**	**Induction of Th2 cytokines; infiltration and activation of pathogenic eosinophils**
**Spontaneous, intestinal fibrosis**	**SAMP1/YitFc mice**	**Blockade of IL-33 by anti ST2 antibody administration**	**Pathogenic**	**Induction of Th2 cytokines; increased pro-fibrotic gene expression**
**Liver**				
**Chemically-induced liver fibrosis**	**Carbon tetrachloride liver intoxication**	**Exogenous IL-33 administration**	**Pathogenic**	**Th1→Th2 switch; pro-fibrogenic cytokine production**[46]
**Heart**				
**Mechanically-induced cardiac hypertrophy and fibrosis**	**Pressure overload by transverse aortic constriction**	**Interruption of IL-33 signaling by genetic deletion of ST2**	**Protective**	**Regulation of NF-kB activation; reduction of macrophage infiltration; Inhibition of angiotensin II and phenylephrine-induced cardiomyocyte hypertrophy**[27]
**Lung**				
**Antitumor antibiotic-induced lung fibrosis**	**Bleomycin-induced lung fibrosis**	**Blockade of IL-33 by sST2 administration**	**Pathogenic**	**Th1→Th2 switch**[28]
**Joints**				
**Rheumatoid arthritis phenotype**	**Collagen-induced arthritis**	**Interruption of IL-33 signaling by genetic deletion of ST2 Exogenous IL-33 administration Bone marrow-derived mast cell adoptive transfer**	**Pathogenic**	**Activation of pathogenic mast cell**[18]
		**Blockade of IL-33 by anti-ST2 antibody administration**	**Pathogenic**	**Induction of IFNγ and IL-17**[17]
**Skin**				
**Skin fibrosis**	**BL/6 mice**	**Exogenous IL-33 administration**	**Pathogenic**	**Accumulation of eosinophils, CD3**^+^**lymphocytes, F4/80+ mononuclear cells; increased IL-13 mRNA expression**[47]

Dextran sodium sulphate (DSS)-induced colitis represents a T cell-independent, chemically induced model of epithelial damage and acute inflammation, primarily driven by innate immune responses. Studying the period immediately after DSS administration (recovery phase) is a useful way to also evaluate mechanism(s) of epithelial repair and mucosal healing. Interestingly, investigation into the role of IL-33 in the development of colitis using this model has generated mixed results, and likely reflects the dichotomous roles of IL-33 in both inducing inflammation as well as promoting epithelial restitution/repair and mucosal healing.

DSS administration to IL-33-deficient mice resulted in less severe colitis than in wild-type (WT) controls, with decreased granulocyte infiltration [42], while exogenous administration of IL-33 to DSS-treated mice further aggravated colitis and induced influx of neutrophils [44], suggesting a pathogenic role of IL-33, at least in an acute inflammatory setting. Although it is unclear as to what factor(s) precisely regulate IL-33 in the gut, it has recently been shown that severe colonic inflammation with a marked increase in IL-33-producing macrophages results after DSS administration to mice expressing a truncated form of the receptor for TGFβ, supporting a pathogenic function for IL-33 during acute colitis and indicates a direct effect of TGFβ on macrophages to limit IL-33 expression [48]. Imaeda *et al*. also reported an exacerbation of DSS-induced colitis upon treatment with IL-33, hypothesized to occur by IL-33-dependent induction of pathogenic Th2 cytokines; although in the same mice, IL-33 restored goblet cells that were found to be depleted in IL-33-untreated mice [43]. In addition, during the recovery phase of DSS-induced colitis, while weight recovery was markedly delayed in IL-33 deficient mice, no significant difference in colonic inflammation was observed between these mice and WT littermates [42]. The authors propose that in this particular model, IL-33 plays an important role in driving acute, innate immune responses, but is dispensable in the maintenance of chronic intestinal inflammation. Alternatively, the possibility exists that the delayed weight recovery observed in IL-33-deficient mice, but not in WT littermates, is due to the lack of IL-33-driven epithelial regeneration and restoration of barrier function leading to a dampened ability for mucosal healing.

In fact, as opposed to their results obtained from IL-33 treatment in acute DSS colitis, Groβ *et al*. showed that IL-33 administration during repeated, chronic cycling of DSS caused a reduction of colitis, suppressed interferon gamma (IFNγ), and decreased bacterial translocation [44], supporting a protective role of IL-33 that the authors suggest may occur by switching from Th1- to Th2-driven immune responses. These results are supported by a recent study using the trinitrobenzene sulfonic acid (TNBS)-induced model of colitis [45]. This model represents another chemically induced model of colonic inflammation that has been reported to elicit Th1 immune responses, particularly if performed using a chronic protocol [49]. Although the aforementioned study utilized an acute, four-day protocol, exogenous administration of IL-33 was shown to ameliorate TNBS-induced colitis and induce the production of Th2-type cytokines [45]. In addition, the protective effect of IL-33 was diminished after depletion of T-regulatory cells (Tregs). The authors propose that, mechanistically, IL-33 has an indirect effect on the development of Foxp3^+^ Tregs by increasing the expression of epithelial-derived thymic stromal lymphopoietin (TSLP) and retinoic acid, which promotes the activation of CD103^+^ dendritic cells [50] and leads to the induction of Foxp3^+^ Treg development [51]. The ultimate IL-33-induced expansion of Foxp3^+^ Tregs facilitates the observed decrease in the severity of colitis.

Using a spontaneous murine model of Th1/Th2-driven enteritis, that is, SAMP1/YitFc (SAMP) strain, IL-33 expression patterns in the gut mucosa and within the systemic circulation of IBD patients, were confirmed [25]. Chronic intestinal inflammation localized to the terminal ileum in SAMP mice occurs spontaneously, without chemical, genetic or immunologic manipulation, and is characterized by an early Th1 immune response and a late, chronic phase of disease dominated by Th2 cytokines [52,53]. IL-33 gut mucosal tissue levels in SAMP mice were shown to progressively increase over time and demonstrated a positive correlation with ileal inflammation, with epithelial cells exclusively expressing full-length IL-33 [25]. Although the precise, mechanistic role of IL-33 has not yet been addressed in the SAMP model, preliminary studies blocking IL-33 signaling by administration of an antibody against ST2 indicate a pathogenic role during the chronic phase of disease development [54,55]. In fact, neutralization of IL-33 interfered with the massive influx of eosinophils into the gut mucosa [55] and potently decreased fibrosis and fibrogenic gene expression [54], characteristic of SAMP mice. Interestingly, although blockade of IL-33 had a significant effect on decreasing the overall severity of ileal inflammation in SAMP mice, the magnitude of this reduction was approximately 30 %, which may reflect a need for optimizing treatment dosage or alternatively, represents an opposing effect of interfering with epithelial repair and mucosal healing. Investigation is further warranted to study the role of IL-33 during the early, acute phase of SAMP ileitis, as well as the specific role of epithelial-derived IL-33 and IL-33's direct effects on the intestinal epithelium.

The hypothesis that innate-type cytokines often possess dichotomous functions within the gut mucosa has been alluded to earlier. This concept is most strongly supported by members of the IL-1Family of cytokines in the pathogenesis of IBD (Table 2) [23-26,40,41,56-63], where the same cytokine can possess both classic proinflammatory properties as well as protective, anti-inflammatory functions, which is dependent primarily on the presence of receptor-bearing cells during a particular setting. As such, aside from the established proinflammatory properties of IL-1α, IL-1β, IL-18 and their downstream signaling molecules, such as NF-κB and MyD88, emerging evidence indicates that the same proteins are necessary for the maintenance of mucosal homeostasis by effectively handling microbiota, as well as by protecting and restoring the integrity of the epithelial barrier [64-66]. The current literature regarding the role of IL-33 in IBD is at present ambiguous, at best; however, it may reflect another example of an innate-type cytokine that possesses multiple functions depending on the immunological status and genetic susceptibility of the host. While one of the first observations of IL-33-dependent epithelial proliferation and mucus production in the gut [5] suggests the promotion of mucosal repair and healing, dysregulated or uncontrolled IL-33 production may lead to the more pathogenic features characteristic of IBD, including epithelial barrier dysfunction, chronic, relapsing inflammation, and formation of fibrotic lesions.

**Table 2:** Speculative role of IL-1 cytokine family members in tissue repair and fibrosis during inflammatory bowel disease (IBD)

**Common name**	**IL-1 Family name**	**Ligand-binding chain**	**Disease association**	**Speculative role in tissue repair and fibrosis**
**IL –1α**	**IL –1F1**	**IL –1R type I**	**CD**[56,57]**, UC**[56,57]	**Protective during early phase of inflammation**[41]
**IL–1β**	**IL –1F2**	**IL –1R type I**	**CD**[56,57]**, UC**[56,57]	**Protective during early phase of inflammation**[41]
**IL –1Ra**	**IL –1F3**	**IL –1R type I**	**UC**[58-60]	**Potential dual role**[41]
**IL–18**	**IL-1F4**	**IL-18Rα**	**CD**[61,62]	**Protective during early phase of inflammation**[41]
**IL–36Ra**	**IL –1F5**	**IL 1Rrp2**	**Unknown**	**Unknown**
**IL–36α**	**IL–1F6**	**IL–lRrp2**	**Unknown**	**Unknown**
**IL –37**	**IL–1F7**	**IL–18Rα**	**Unknown for human IBD, antagonist for DSS colitis**[63]	**Protective (correlates with breakdown of intestinal barrier)**[63]
**IL-36β**	**IL-1F8**	**IL-1Rrp2**	**Unknown**	**Unknown**
**IL-36γ**	**IL-1F9**	**IL-1Rrp2**	**Unknown**	**Unknown**
**IL–38**	**IL–1F10**	**IL 1Rrp2**	**Unknown**	**Unknown**
**IL- 33**	**IL –1F11**	**ST2**	**UC**[23-26]	**Protective**[40,41]

### Emerging role of IL-33 in fibrosis

The concept that fibrosis occurs as the end result of dysregulated wound healing is well established. Th2 cytokines, such as IL-4 and IL-13, have been reported to be important factors in the development of fibrosis [67-69] and collagen synthesis [70,71]. Relevant to the present review, IL-33 was originally described as a potent inducer of the Th2 cytokines, IL-4 and IL-13, by CD4^+^ T lymphocytes [5], and administration of recombinant IL-33 into WT mice increased local tissue levels of IL-4, IL-5, and IL-13 in the liver [46]. In fact, expression of IL-33 and its receptor/coreceptor, ST2/IL-1RAcP, has been associated with both murine and human liver fibrosis, with a direct correlation between IL-33 and ST2 levels, but not IL-1RAcP, and the severity of fibrosis. Moreover, IL-33 levels have been shown to closely correlate to collagen synthesis in both mouse and human livers during chronic injury [46]. Liver endothelial and hepatic stellate cells (HSCs) constitute the major sources of IL-33 in liver fibrosis, and the increase in IL-33 mRNA levels are likely due to the activation and proliferation of HSCs and/or the increased number of endothelial cells [46]. T lymphocytes appear to be a primary target for soluble IL-33 as they represent a large percentage of ST2-positive cells that are present in human fibrotic livers [72] and are crucial for the development of fibrosis [5,67-69]. As such, it is likely that IL-33-dependent fibrogenesis in the liver involves the production of IL-33 by activated HSCs that subsequently act on ST2-bearing T cells, stimulating profibrogenic Th2 cytokines to initiate the process of fibrosis [46].

IL-33 is also considered to be a novel factor involved in the pathogenesis of pancreatic fibrosis as a consequence of chronic pancreatitis [73]. Fibrosis of the pancreas is one of the representative histopathologic findings in cases of chronic pancreatitis where pancreatic myofibroblasts play a crucial role. Nishida *et al*. recently reported that human pancreatic myofibroblasts are both a source of IL-33 as well as express the IL-33 receptor complex of ST2L/IL-1RAcP [73]. In this study, IL-33 secretion is potently induced through a mitogen-activated protein kinase (MAPK)-dependent activator protein-1 (AP-1) pathway by IL-1β, TNF, and LPS, while ST2L expression is upregulated by IL-4 and IFNγ by activation of STAT6 and STAT1, respectively. IL-33 was shown to enhance the expression of proinflammatory mediators in IL-4- and IFNγ-pretreated pancreatic myofibroblasts and to stimulate the proliferation and migration of these cells [73].

Although the role of IL-33 has not yet been fully investigated in the pathogenesis of intestinal fibrosis, several lines of evidence indicate that the IL-33/ST2 axis may represent an important mediator in this process. Within the gut mucosa, SEMFs have been reported as a primary source of IL-33, specifically in UC patients where they are situated below ulcerative mucosal lesions [24,40]. In fact, Sponheim *et al*. observed that a prominent feature of IBD-associated IL-33 expression is the accumulation of both fibroblasts and myofibroblasts in ulcerations of UC lesions [40]. Although, the association and localization of IL-33-producing SEMFs with mucosal ulcerations suggests an important role in wound healing, one cannot rule out its potential role in gut-associated fibrosis, particularly in the setting of cycling of chronic tissue damage and repair, characteristic of IBD. In addition, the GI tract represents the largest organ interfacing with the external environment and interacting with luminal antigens, infectious agents, and other components of the gut microbiota. Interestingly, toll-like receptor (TLR)3, TGFβ, and mechanical strain have been shown to promote the development of myofibroblasts from connective tissue fibroblasts in wound repair [74,75], wherein fibroblasts acquire α-smooth muscle actin-containing stress fibers in order to successfully achieve wound contraction and healing [76]. Contrary to its effects on gut-associated macrophages [48], TGFβ was shown to boost TLR3-dependent induction of IL-33 in cultured fibroblasts [40]. TLR3 signals the presence of virus-derived double stranded RNA, but mRNA released from damaged or necrotic epithelial cells can also lead to TLR3 activation, which has been shown to protect against DSS-induced colitis [77]. Based on its similarity to the physiological wound healing seen in the skin [40], the recruitment of IL-33-positive fibroblasts to ulcers is considered a protective process driven by the loss of the epithelial barrier and by the presence of the gut microflora that acquires a critical role in modulating mucosal healing. Interestingly, subcutaneous administration of IL-33 to WT mice has been shown to lead to the accumulation of inflammatory cells and the development of skin fibrosis, which is dependent on IL-13 and the presence of eosinophils [47]. Eosinophils and their degranulation have, in fact, been implicated in the process of fibrosis in several organ systems, including the GI tract [78]. Although a direct correlation has not yet been established between eosinophilia and fibrosis in the SAMP model of chronic intestinal inflammation, preliminary studies have demonstrated that IL-33 represents a critical factor important in the development of both processes [54,55].

### Potential link between IL-33 and GI-related cancer

Based on the established role of IL-1Family members, including IL-1β and IL-18, in GI-related cancers, the possibility exists that IL-33 can likewise play an important role in GI-associated tumor formation. In fact, a recent study has reported elevated IL-33 levels in the serum of gastric cancer patients that correlated with several poor prognostic factors, including depth of invasion, distant metastasis, and advanced stage, but not with the classic tumor markers, CEA and CA 19–9 [79]. Of note, however, no significant difference in IL-33 expression was found between four gastric cancer cell lines and the normal gastric cell line, GES-1, which may indicate that IL-33 expression can either be modulated by local environmental factors and/or produced by other cells responding to gastric cancer epithelial cells. As such, the initial observation of increased, circulating IL-33 levels in gastric cancer patients may be related to the progression of the cancer. In addition, based on IL-33's ability to shift host immune responses to a Th2 phenotype, downregulation of tumor-specific immune responses can occur by inhibiting tumor antigen presentation [80,81]. From this point of view, IL-33 may represent one of the effective weapons tumor cells utilize in order to create an ideal environment for obtaining optimal growth conditions. IL-33 has also been reported to be elevated in lung tissues of K-ras transgenic mice [82], which are prone to tumorigenesis and cancer, in endothelial cells from several distinct human tumors, and in cancer epithelial cell lines [4,83]. Furthermore IL-33 is able to activate NF-κB that has been implicated in the development of several types of human cancers [84,85], and is a common transcription factor involved in signaling of several IL-1Family members. Finally, in a recent report, suppression of breast cancer progression and metastasis was observed in a murine breast cancer model wherein mice lacked the IL-33 signaling receptor, ST2 [86], further supporting the role of the IL-33/ST2 axis in tumor formation and the progression of cancer.

## Conclusions

**Figure 1:**
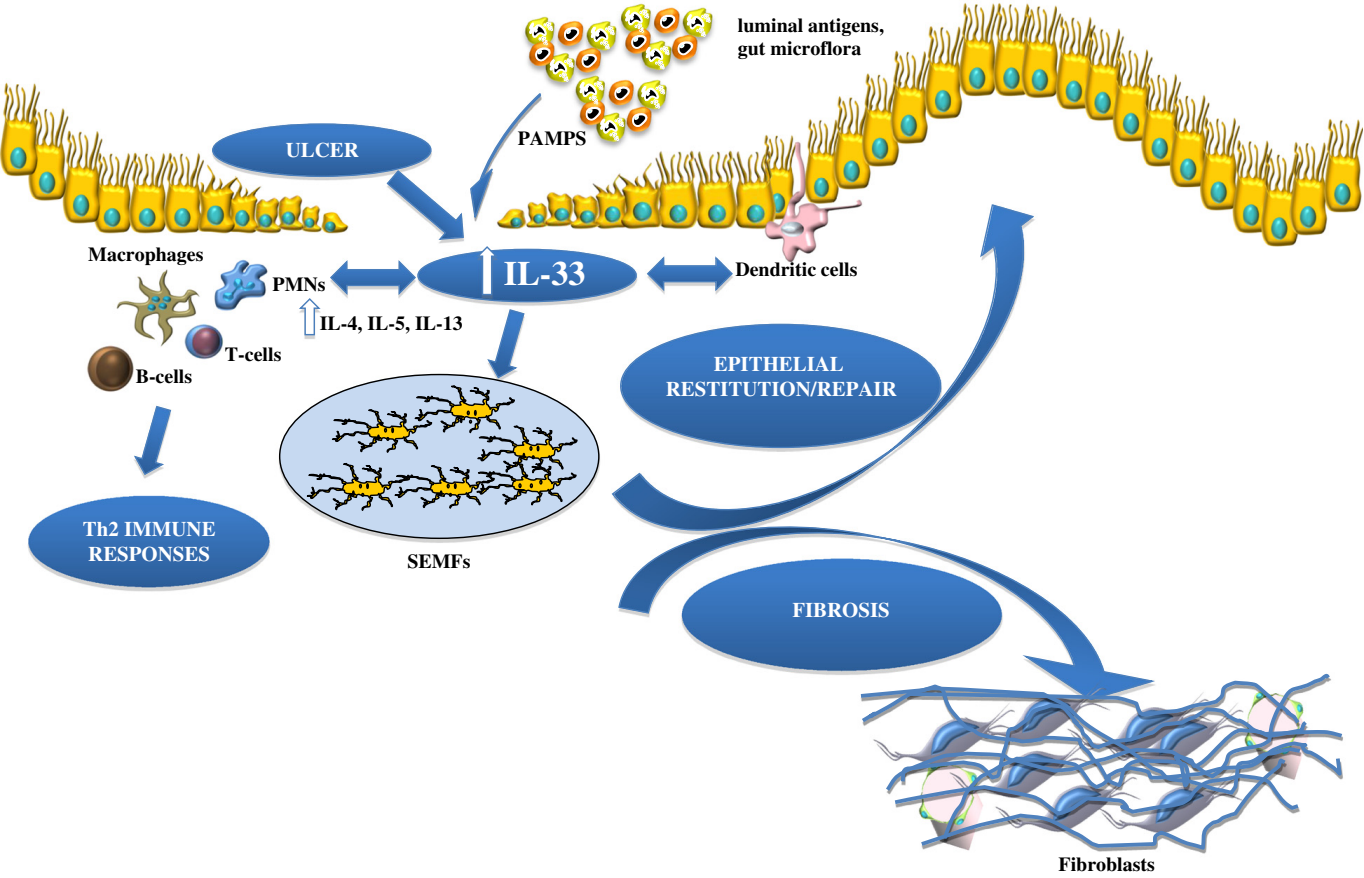
**Working hypothesis of IL-33's dichotomous role in the gut mucosa.** Damage of the epithelium (for example, ulcer formation) and other proinflammatory stimuli, including pathogen-associated molecular patterns (PAMPs) derived from luminal antigens and the local intestinal microflora, induce intracellular IL-33 expression that is subsequently released by necrotic intestinal epithelial cells (IECs) as a potential alarmin. Depending on the presence and abundance of ST2-bearing target cells and the phase of disease process, IL-33 may have very different effects within the gut mucosa. IL-33 may act on various immune cell populations, including macrophages, and T and B cells, eliciting proinflammatory effects and promoting Th2 immune responses. Concomitantly, IL-33 can also induce epithelial proliferation and repair and overall wound healing by acting directly or indirectly on IEC and subepithelial myofibroblast (SEMFs). Alternatively, chronic mucosal damage, granulomatous inflammation, and dyregulated activation of mesenchymal cells, such as SEMFs and fibroblasts, can lead to fibrosis and the formation of intestinal fibrotic lesions.

The present review provides evidence that, aside from its central role as a classic proinflammatory cytokine promoting Th2 immune responses, IL-33 also plays a critical role in modulating epithelial repair, mucosal healing, and fibrosis in the GI tract during normal gut homeostasis and in the setting of chronic intestinal inflammation (summarized in Figure 1). Although less developed, a potential association between IL-33/ST2 in the process of tumorigenesis and GI-related cancer may also exist. Based on this new information, novel pathogenic hypotheses can be formed that have important translational implications in regard to the prevention and treatment of chronic intestinal inflammation, including CD and UC. IL-33 may also play a potential role in sustaining tumor growth, by modulating host immune responses against tumor cells and/or in the recruitment of SEMFs to support their growth. Further mechanistic studies will clarify the precise physiologic and pathophysiologic role of IL-33 in the GI tract.

## Abbreviations

AP-1: activator protein-1; CD: Crohn’s disease; DSS: dextran sodium sulphate; GALT: gut-associated lymphoid tissue; GI: gastrointestinal; HSC: hepatic stellate cell; IBD: inflammatory bowel disease; IEC: intestinal epithelial cells; IFNγ: interferon gamma; IL: interleukin; LPS: lipopolysaccharide; MAPK: mitogen-activated protein kinase; SAMP: SAMP1/YitFc; SEMF: subepithelial myofibroblast; TGFβ: transforming growth factor beta; TLR: toll-like receptor; TNBS: trinitrobenzene sulfonic acid; TNF: tumor necrosis factor; Treg: T-regulatory cell; TSLP: thymic stromal lymphopoietin; UC: ulcerative colitis; WT: wild-type.

## Competing interests

The authors declare they have no competing interests.

## Authors’ contributions

LL did most of the research and writing. FS assisted with the editing. TTP conceived the original concept for the review, assisted with the research, and performed the editing. All authors have read and approved the final manuscript.
